# Ubiquitination and deubiquitination as critical modulators of NSCLC tumorigenesis and drug resistance

**DOI:** 10.1007/s12672-026-04455-w

**Published:** 2026-01-31

**Authors:** Prasanna Srinivasan Ramalingam, Mohammad Afzal, Manjunath Mirle Rekha, Samir Sahoo, Surya Nath Pandey, Chandana Maji, Kavita Goyal, Haider Ali, Sachin Kumar Singh, Gaurav Gupta, Md. Sadique Hussain, Purushothaman Balakrishnan, Sivakumar Arumugam

**Affiliations:** 1 Protein Engineering Lab, School of Biosciences and Technology, Vellore Institute of Technology, Katpadi, Vellore, 632014 India; 2 Department of Pharmaceutical Sciences, Pharmacy Program, Batterjee Medical College, Jeddah, 21442 Saudi Arabia; 3 Department of Chemistry and Biochemistry, School of Sciences, JAIN (Deemed to Be University), Bengaluru, Karnataka India; 4 Department of General Medicine, IMS and SUM Hospital, Siksha ‘O’ Anusandhan (Deemed to be University), Bhubaneswar, India; 5 Department of Pharmacology, Teerthanker Mahaveer College of Pharmacy, Teerthanker Mahaveer University, Moradabad, India; 6 Noida Institute of Engineering and Technology (Pharmacy Institute), 19 Knowledge Park 2, Greater Noida, India; 7 Sharda University, Greater Noida, India; 8 Department of Pharmacology, Kyrgyz State Medical College, Bishkek, Kyrgyzstan; 9 School of Pharmaceutical Sciences, Lovely Professional University, Phagwara, India; 10 Centre for Research Impact & Outcome, Chitkara College of Pharmacy, Chitkara University, Rajpura, Punjab India; 11 Uttaranchal Institute of Pharmaceutical Sciences, Uttaranchal University, Dehradun, 248007 Uttarakhand India; 12 TanBio R and D Solution, Thiruvarur, 610103 TamilNadu India

**Keywords:** Ubiquitination, Deubiquitination, Non-small cell lung carcinoma, Protein stability, Ubiquitin ligases

## Abstract

Ubiquitination and deubiquitination are biological mechanisms that including the addition and removal ubiquitin molecules from proteins, which are associated with NSCLC. NSCLC is the most common kind of lung cancer, and treatment resistance and cancer development are linked to these alterations. Ubiquitination incorporates proteins by a particular enzyme class, and deubiquitination removes ubiquitins. In NSCLC, this modification is detrimental to cellular function and affects tumorigenesis, apoptosis, and signaling pathways. Conversely, deubiquitination refers to the process through which the ubiquitins are removed from the proteins so that they can perform their functions properly. These processes can be considered therapeutic in some sense, as regulating both can result in an effective therapy for NSCLC. Many studies have been carried out to find potential areas where further drug research could be focused. This study examines the functions of ubiquitination and deubiquitination in NSCLC pathogenesis and resistance, focusing on their mechanisms, consequences, and therapeutic possibilities.

**Graphical Abstract:**

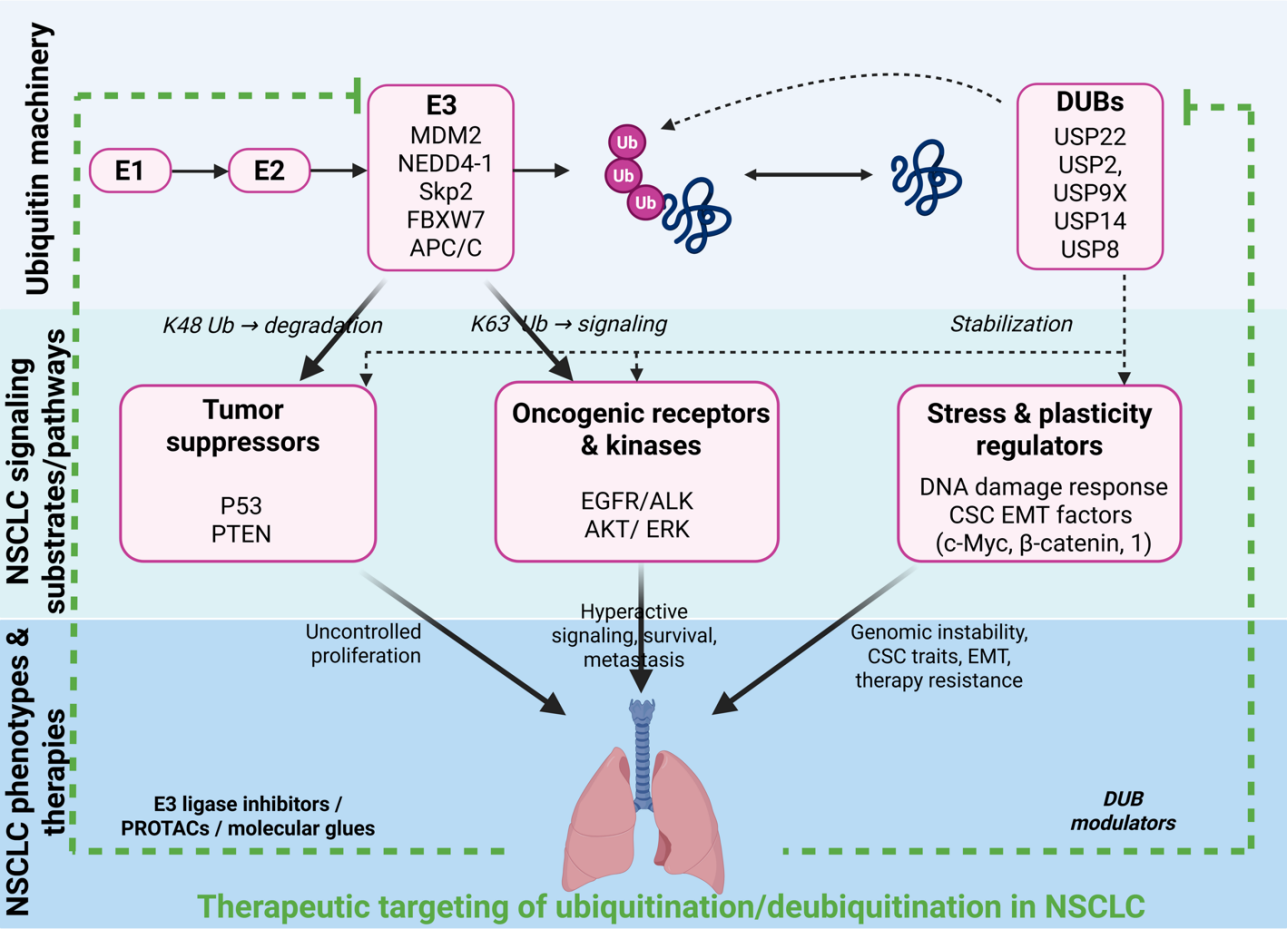

## Introduction

Non-small cell lung cancer (NSCLC) account for more than 85% of all lung cancer cases and encompasses three main histological subtypes: squamous cell carcinoma (SCC), adenocarcinoma, and large cell carcinoma [1, 2]. SCC, often linked with smoking, develops from the squamous epithelium of the bronchial areas of the lung [3]. These cancerous cells include keratinization and intracellular bridges [4], which tend to arise in the central lung regions, particularly around the bronchus [5]. SCC usually presents with well-differentiated lesions, whereby the tumor cells have some degree of structural organization and usually organize themselves into layers resembling human skin [6]. Adenocarcinoma, which is prevalent among non-smokers, originates from a glandular layer of secretion-bearing cells in the outer perspective of the lungs [7, 8]. A characterized histological pattern of this subtype includes acinar, papillary, bronchioloalveolar, and solid formations; it also often has well-known mutations, such as mutations in the EGFR gene, for which it is more sensitive to targeted gene therapy [9, 10]. Large cell carcinoma, however, presents no specific histopathological difference from SCC and adenocarcinoma [11]. NSCLC is a rapidly growing and difficult-to-treat form of NSCLC characterized by large, undifferentiated cells. It is the fastest-growing and hardest-to-treat tumor worldwide [12]. Variables leading to the elevated prevalence and death instances of NSCLC involve tobacco use, exposure to environmental toxins, and genetic susceptibility [13, 14]. Other contributing factors include exposure to chemical substances, air pollution, and genetic predisposition [15]. Unfortunately, early-stage NSCLC is often asymptomatic, resulting in late-stage diagnosis when therapeutic choices are restricted. However, recent breakthroughs in understanding the molecular pathology of NSCLC have led to improvements in targeted therapies [16, 17].

Ubiquitination, a post-translational modification that can determine protein stability and functioning, is one of the major molecular mechanisms involved in NSCLC [18]. Ubiquitin is a small regulatory protein, and the whole process of Ub conjugation in this manner consists of a cascade of enzymes for synthesis, including E1 enzyme (for activation), an E2 conjugating enzyme, and an E3 ligase, which promotes covalent attachment to the substrate protein [19, 20]. During this process, many of the cellular fates could be ruled out, from protein degradation through proteasome to vesicular movement in the cell or activation of some signaling pathways and all those with great consequences for cell function and cancer progression [21]. Conversely, the cleavage of ubiquitin from proteins by deubiquitinating enzymes (DUBs), i.e., deubiquitination, is also important to maintain protein homeostasis [22]. A fraction of ubiquitin molecules are recycled via deubiquitination to govern normal protein regulation and maintenance of cellular homeostasis [23]. For cancer, especially NSCLC, dysregulation of these pathways can lead to unlimited cell proliferation, prevent apoptosis and increase tumour aggressiveness [24, 25]. The deregulation of these processes in cancer, especially NSCLC, may result in continuous cell growth and accumulation along with resistance to apoptosis, ultimately resulting in tumor aggressiveness [26].

Our main finding, on which we build in this review, is that dysregulated ubiquitination/deubiquitination is a systems-level hub in NSCLC, which rewires oncogenic signalling dynamics, tumor cell plasticity and stemness, and intrinsic and acquired resistance to conventional and targeted therapies. Specifically, we answer three improtant questions: (1) how the ubiquitin-proteasome system (UPS) remodels core pathways (EGFR/ALK, PI3K/AKT/mTOR, NF-κB, and Wnt/β-catenin) to drive NSCLC development; (2) how distinct classes of E3 ligases and deubiquitinases (DUBs) converge on overlapping signs of NSCLC, such as evasion of apoptosis, DNA-damage resistance and metabolic adaptation; and (3) how these mechanistic understandings can be translated into treatment approaches, such as DUB and E3 inhibitors, PROTACs and combination regimens with chemotherapy, targeted agents and immunotherapy. Having arranged the literature in lines with these questions and not by enzyme in isolation, we hope to offer a composite framework which reveals the nodal vulnerabilities and the remaining gaps in the field.

Significantly, new literature suggests that ubiquitination and deubiquitination should not be considered homogenous and not context-dependent regulators in lung cancer, and they are highly influenced by the genetic and histological context of NSCLC. Dependence on particular E3 ligases and DUBs as well as recruitment of various stress-adaptation modules is also different in EGFR-mutant, ALK-rearranged, KRAS-mutant and smoking-associated tumours, and adenocarcinoma versus squamous cell carcinoma subtypes. Simultaneously, the growing usage of immune checkpoint inhibitors and targeted therapies has demonstrated that the UPS components can augment or restrain the immune evasion and adaptive resistance [27]. By noting these context-specific and subtype-associated regulatory subsets throughout the review, we expect to resonate ubiquitin biology with the existing discussion on how molecularly defined NSCLC subgroups may have specific therapeutic vulnerabilities of the ubiquitin system. The rest of this review proceeds as a story in these three leading questions. We will begin by translating the general concepts of ubiquitination and deubiquitination to their effects on fundamental oncogenic pathways in NSCLC. Then we think how specific E3 ligases and DUBs can regulate shared targets of stress responses, genomic consistency as well as cell plasticity. Last but not the least, there is the discussion on how these mechanistic understanding can be applied to therapeutic interventions and resistance-altering list in NSCLC.

## Ubiquitination and deubiquitination

To understand how these processes contribute to NSCLC, it is first necessary to outline the core enzymatic machinery and modes of ubiquitination and deubiquitination. One important post-translational modification is ubiquitination, which controls the stability and functioning of proteins in the cytoplasm and even suppresses them in particular cellular compartments before directing them to the proteasome for destruction [28]. Ubiquitin is a 76-amino acid protein that is attached to a substrate during this process [29]. The ubiquitination system can be regulated by a series of three types of enzymes for their assay in an orderly manner. At the initial step, the E1 enzyme triggers ubiquitin in an ATP-dependent reaction by forming a high-energy thioester bond with ubiquitin [30–32]. Ubiquitin is then moved to the E2 enzyme. However, the E3 ligase is a high specificity binding protein to misfolded proteins in response & then again transfer of Ub to lysine residues of substrate protein [33]. The proposed enzymatic cascade must also be conceptualized as a signalling platform in which ubiquitin linkages and DUB editing have been selectively fine-tuned to oncogenic and stress-response cascades, even in the context of NSCLC.

Ubiquitination exists in different modes, thus leading to different cellular outcomes [34]. Monoubiquitination represents the addition of only one ubiquitin component to the substrate protein. In contrast, multi-monoubiquitination implies that several ubiquitin molecules are bound on different lysine present in the target protein [35]. In polyubiquitination, ubiquitin chains are formed on the substrate. The precise fate of the tagged protein will be governed by what type of ubiquitin chain linkage is created [36]. K48-linked polyubiquitin chains usually lead to protein breakdown by the 26 S proteasome and are crucial for the repair of cellular protein homeostasis [37–39]. In addition, K63-linked chains participate in non-degradative procedures such as signal transduction, DNA repair, and endocytosis7 [40, 41], demonstrating the versatility of ubiquitination to fulfill its roles in cellular functions.

Various ubiquitin linkage produces diverse mechanistic results. K48- and K11-linked chains normally carry out proteasomal degradation of substrates [42] but K63- and M1-linked chains serve as non-proteolytic frameworks assembling signalling complexes [43]. This switching effect of the same substrate between fates can be affected by the type of its linkage: K48-linked PTEN or p53 undergoes quick degradation, whereas K63-linked EGFR, AKT, and RIP1 endorse long-term oncogenic signaling [44, 45]. Therefore, chain type, length and branching which form the ubiquitin code of an individual play a crucial role in cell survival, growth, and stress adaptation in NSCLC.

As deubiquitination functions as the opposing process to ubiquitination, it acts to maintain a balanced regulation of protein turnover and activity. DUBs mediate this procedure by removing ubiquitin either from target proteins or dismantling ubiquitin chains; thus, they prevent proteasomal degradation [46]. Besides recycling ubiquitin, deubiquitination isoenzymes also play a role in the modulation of protein activity, localization, and function [23]. There are nearly 100 DUBs known in human tissues, which can be classified into distinct subclasses: ubiquitin-specific proteases (USPs), ubiquitin carboxyl-terminal hydrolases (UCHs), ovarian οumor domain DUBs (OTUs), Josephin domain DUBs, and JAB1/MPN/MOV34 metalloenzymes [47, 48]. Therefore, the richness of ubiquitin chain structures, as well as the range of DUB families, offer the molecular framework in the systems level control that we subsequently see within NSCLC, where identical enzymatic modules mediate the activity of several hallmarks, including proliferation, survival and response to therapy.

**Fig. 1:**
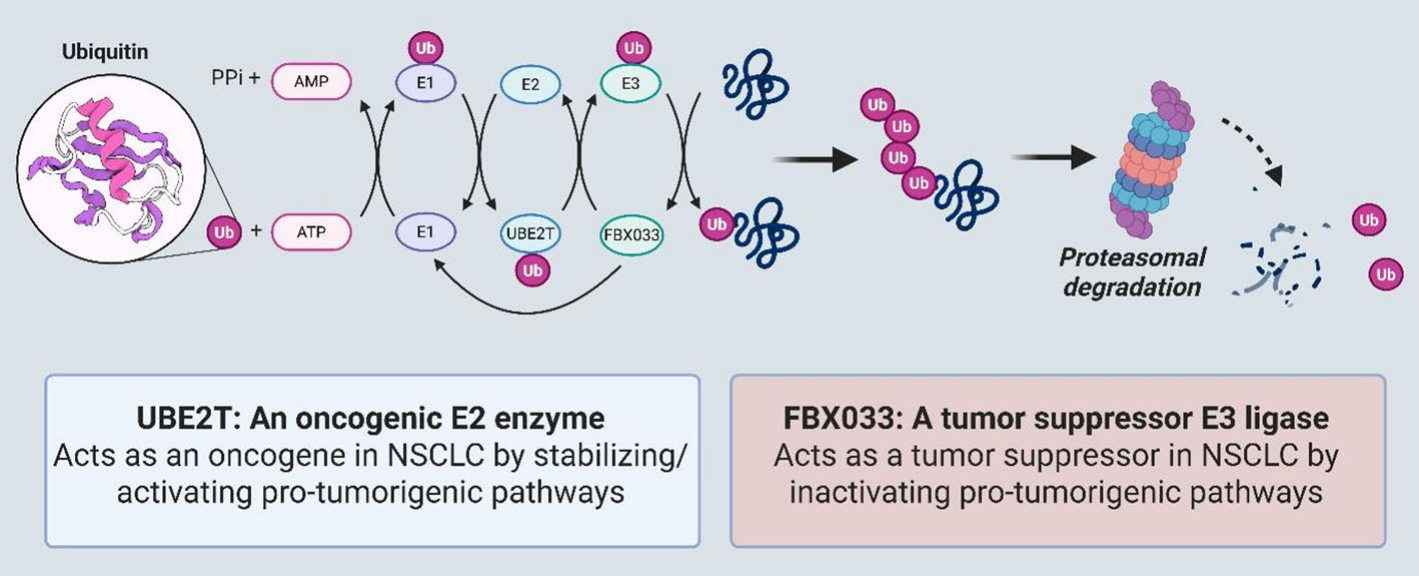
The image illustrates the ubiquitination process, showing the activation and transfer of ubiquitin (Ub) through enzymes E1, E2, and E3, leading to a polyubiquitin chain attached to a protein. Then the ubiquitinated protein gets degraded by the proteosomal degradation processes

The precise regulation of ubiquitination and deubiquitination is necessary for sustaining cellular homeostasis [49]. These processes can be dysregulated and result in diverse disorders, including cancer, neurodegenerative diseases, and immune deficiencies [50]. Cancers overexpress or mutate specific E3 ligases to degrade tumour suppressor proteins and promote uncontrolled cell proliferation. Conversely, should any user gift with DUB exercise get thwarted, this could lead to the downregulation of oncoproteins that regulate cellular proliferation and apoptosis via disruption of major signalling pathways **(**Fig. 1**)** [51]. The updated Fig. 1 illustrates the ubiquitin cascade in detail, showing ATP-dependent activation of ubiquitin by E1, transfer to E2 (with UBE2T), and substrate ubiquitination by representative E3 ligases (MDM2, UBE3C, FBXO33, Skp2 and APC/C), thereby linking the core enzymatic steps directly to NSCLC-relevant pathways and substrates.

In case of NSCLC, the following general principles can be applied to give rise to highly context-dependent landscape where a given UPS component can be either tumor-promoting or inhibiting, based on the substrate and signaling context. In the subsequent discussion, we will thus not dwell too much on a comprehensive list of known E3 ligases and DUBs, but more on how some of these enzymes can be organized into functional modules to shape the biology of NSCLC. Three common themes that are recurring are emphasized by us; (i) oncogenic signaling combinations tuned by UPS; (ii) reinforcing cell-intrinsic stress responses, genome maintenance and metabolism, and (iii) rewiring drug response and resistance. This modular perspective can be used to balance seemingly unrelated observations and create identified mechanistic nodes where therapeutic intervention can possibly create system-wide effects.

## Ubiquitination and non-small cell lung carcinoma

Biologically, the effects of ubiquitination in NSCLC can be classified into three intersecting themes, including; (i) rewiring oncogenic signalling networks, (ii) control of cell-intrinsic stress and genome-maintenance programs, and (iii) regulation of intrinsic and acquired resistance to therapy. Mechanistically, we must consider ubiquitination in NSCLC not as a simple terminal tag but as a dynamic type of code that functions selectively to either enhance or to mutter insecure signaling stimuli. Instead of discussing individually each E3 ligase, it is convenient to think about how panels of ubiquitin-modifying enzymes are used together to remodel a restricted number of ubiquitin signal transduction pathways that regulate tumour behaviour. These hubs are most notable in the PI3K/AKT/mTOR and RAS-RAF-MEK-ERK signaling and NF-ĸB-based inflammatory pathways and cell-cycle checkpoints, and DNA-damage pathways in NSCLC.

In NSCLC, the dysregulated ubiquitin activity alters the degradation and stability of key regulatory proteins, thereby facilitating tumour growth, progression, and metastatic behaviour [52]. This is facilitated by the UPS, which is important for regulating protein homeostasis [53]. E3 ligases specifically label proteins to be broken down with ubiquitin. The resulting poly-ubiquitinated substrates are then guided to the 26 S proteasome for degradation [54]. Consequently, disrupting this system can result in upregulation of oncoproteins or undermining the functioning of tumor suppressor genes, resulting in unregulated cell growth and development of tumors [55].

A significant feature of NSCLC tumorigenesis involves the participation of ubiquitination in driving protein degradation and stability positively or negatively in vital cascades such as PI3K/AKT/mTOR axis and NF-κB [56–58]. These pathways regulate processes crucial for the survival, growth, and metastasis of cancerous cells. For instance, the tumor suppressor PTEN works as a primary negative modulator of PI3K/AKT cascade, and its degradation by overexpressed E3 ligases is a frequent cancer loss-of-function mechanism in NSCLC [59–61]. NEDD4-1 and CHIP are some of the established E3 ligases targeting PTEN. Loss of PTEN, which results in pathway hyperactivation, drives tumor survival and aggressive growth [62]. On the contrary, stabilized oncogenes caused by impaired ubiquitination serve as a check on cancer cell growth and invasiveness of spontaneously arrested tumors, bringing us to paradoxical conclusions in which ubiquitination is pro-carcinogenic or anti-carcinogenic relying on the cellular context [63]. These data demonstrate the convergence of the different E3 ligases, although mechanistically varied, on a comparatively limited set of signalling hubs, which reflects our conception of ubiquitination as a pathway-based rather than enzyme-based driver of NSCLC.

Collectively, the accessible evidence indicates that the oncogenic effect of ubiquitination in NSCLC is pathway-centric instead of enzyme-centric. There is a high level of redundancy as multiple E3 ligases converge on common targets like PTEN, c-Myc, and PI3K/AKT/mTOR or NF-ĸB cascades. CYLD was reported to act as DUBs in NF-ĸB pathway. This overlaps with the reason why even single-enzyme perturbations can cause phenotypes not so large in certain experimental models and why it is important in some situations to develop combinatoric approaches by either attacking multiple UPS components in the same signaling module or synergizing UPS modulation with direct inhibition of downstream kinases or transcription factors.

A series of specific ubiquitinating enzymes and DUBs have been shown to contribute in NSCLC tumorigenesis [63]. USP28, c-Myc stabilizing DUB, which is involved in lung cancer pathogenesis [64–66]. Under normal circumstances, c-Myc and similar oncoproteins are marked for degradation through the proteasome pathway [64]. However, overexpression of USP28, which is common in NSCLC, prevents the degradation of these proteins by recycling ubiquitin, allowing oncoproteins to accumulate and drive tumor progression. The resulting increase in c-Myc levels contributes to the poor clinical outcomes often seen in NSCLC patients [64, 67, 68].

## Deubiquitination and non-small cell lung carcinoma

Deubiquitination catalyzed by DUBs, counterbalances ubiquitination by removing ubiquitin from target proteins and thereby reshaping protein stability and signalling outputs in NSCLC. This mechanism is crucial for controlling protein turnover, ensuring cellular homeostasis, and maintaining protein stability [69]. In NSCLC, deubiquitination has been linked to treatment resistance, as DUBs stabilize several proteins involved in cell viability, division, and apoptosis [70]. By maintaining the stability of these proteins, cancerous cells can hinder the influence of therapy, including chemotherapy, immunotherapy, and targeted therapy, contributing to poor treatment outcomes. DUBs do not act in isolation, but they work in pairs (or networks) with E3 ligases. An individual substrate can also be armed with a degradative (K48) chain and a signaling (K63) chain by different E3s and each protein type chain is then precisely edited by that particular DUB [71]. This regulatory circuit breaker is a combinatorial control element that balances receptor signalling, DNA repair, protection against oxidative-stress, and apoptotic thresholds in NSCLC. In further detail, the examples below may be viewed through the prism of the three above-presented themes: USP9X and USP14 are mainly promoting anti-apoptotic survival signalling, USP8 and associated DUBs are upholding oncogenic receptor and oxidative-stress homeostasis, and OTUD1 and additional genome-stability DUBs are disrupting the responses of DNA-damage and cell-cycle regulation.

Theoretically, DUBs in NSCLC may be clustered based on the stress or survival axis they maintain. There are DUBs (USP9X, USP14) which appear to stabilize anti-apoptotic proteins, counteracting proteasome overload, and apoptotic stress; yet others (USP8, USP10, USP11) maintain oncogenic receptor homeostasis, resistance to oxidative-stress or tolerance to DNA-damage. In this section we highlight how these functional classes overlap over shared phenotypes, chemoresistance, radioresistance, and immune evasion, as opposed to listing the DUBs singly.

Deubiquitination can lead to resistance in NSCLC by stabilizing proteins that promote cell survival and inhibit apoptosis [72], DUB USP9X, which stabilizes the anti-apoptotic protein MCL-1. Under normal circumstances, polyubiquitin chains direct MCL-1 for proteasome destruction [73]. However, USP9X removes these ubiquitin chains, preventing MCL-1 from being degraded [74]. Elevated levels of MCL-1 are associated with tolerance to chemotherapeutic medications such as cisplatin, a commonly used drug in NSCLC treatment [75]. The proteasome-dependent degradation of MCL-1 promotes cell death and, as a result, boosts the efficiency of cisplatin treatment in cancer cells [76]. However, the activity of USP9X attenuates this apoptotic response by increasing MCL-1 stability, resulting in lower efficiency of chemotherapies [77].

Deubiquitination further drives resistance to treatment because under-expression of USP14 will potentiate proteasome inhibitors in cancer cells. They are designed to enhance the breakdown of misfolded proteins, resulting subsequently to apoptosis [78–80]. Under normal conditions, USP14 removes ubiquitin from these proteins, protecting cells from apoptosis normally facilitated by the toxic build-up of specific proteins [81]. Consequently, cancerous cells continue to survive and expand in the presence of proteasome inhibitors, demonstrating that USP14 participates in protecting NSCLC cells from therapeutic interventions [82]. Besides determining chemoresistance in part, deubiquitination can offer a role to the sensitivity of targeted treatments for NSCLC [83]. Another DUB, family member USP8, can enhance oncogenic receptor EGFR and ALK stability by the deubiquitylating of ubiquitin tags as well. This stabilization preserves the activity of critical mitogenic and pro-survival signalling pathways in cancer cells [84–86]. The aggregate of these results will confirm a model according to which DUBs can act as rheostats that refine the levels and periods of survival signaling in NSCLC. Notably, the same DUB frequently stabilizes several substrates in various pathways (e.g. survival proteins, DNA-repair factors, metabolic enzymes), a fact that may also help comprehend the wide-ranging resistance phenotypes of its dysregulation. This pleiotropy is a two-sided sword: it is associated with the risk of on-target toxicity, but implies that a success of targeting a single DUB can debulk multiple circuit breakers of resistance.

A significant unresolved problem is the context-dependent role of a number of DUBs in NSCLC. As a case study, USP9X has been identified to stabilize anti-apoptotic proteins and help sustain alternative NSCLC models [87], but other evidence proposes tumor-restrictive capabilities by carrying out genome integrity or control of particular substrates [88]. Likewise, the USP17 has been found to drive proliferation, migration, and EMT and also differentiation-related programmes in certain genetic backgrounds [89]. These seemingly conflicting functions probably indicate variations in oncogenic driver mutations, tumor subtype, the microenvironment and the range of substrates expressed in a particular context. It is important to acknowledge that this dependence on context is a warning not to simplify the classification of single DUBs as being either uniformly oncogenic or tumor-suppressive in NSCLC. Collected, these observations point towards the idea that DUBs are context-specific rheostats, not isolated switches, of systems-level hub-based model of NSCLC signalling, which is a significant observation of our systems-level hub model.

## Clinical implications for ubiquitin-regulated signaling pathways and therapeutic targets

Targeting ubiquitination and deubiquitination in NSCLC cannot be done in isolation of the signaling pathways that these enzymes control. We here thus mention ubiquitin-modifying enzymes specifically in their pathway context: Sect. 5.1 discusses specific DUBs and associated enzymes as targets of therapeutic interest, Sect. 5.2 provides an overview of the key ubiquitin-regulated signaling pathways which are the therapeutic nodes, and Sect. 5.3 identifies ubiquitin-modifying enzymes as drivers of treatment resistance. This unified organisation connects targets directly to the channels where they are operating.

### Ubiquitin-specific peptidase as therapeutic targets in NSCLC signaling networks

There are node regulators and some USPs present within the DUBs family which connect chromatin state, cell-cycle control, and oncogenic signaling with clinical behaviour in NSCLC. Instead of individual USP, it is informative to take the group of USP5, USP22, USP28, USP44, USP 49, and others to influence three important aspects of NSCLC biology: management of stem-like states, control of p53-dependent checkpoints, and regulation of pro-survival cascades.

USPs offers crucial functions in NSCLC by facilitating cancer progression and therapeutic resistance, which has attracted a lot of recent attention. A major player is USP22, which controls histone H2B monoubiquitination (H2Bub1) [90]. The deubiquitination of H2Bub1 is associated with lung cancer cell differentiation reduced, the CSCs properties are high, and the malignancy increased [91, 92]. Zhang et al. showed that USP22 is overexpressed in lung cancer tissues, and this is corelated with poor patient survival as well as high-grade tumors. A comparison study suggested that depletion of USP22 using a genetic approach inhibited tumor proliferation, metastasis, and angiogenesis but sensitized to cisplatin treatment and irradiation, leading to the prolonged survival of lung-tumor-bearing mice [93]. Similarly, Ning et al. discovered that USP22 levels are higher in NSCLC tissues with a high degree of tumor size, lymph node metastasis, and reduced patient survival, which suggested its possibility as an early-stage NSCLC prognostic marker​ [94].

**Fig. 2:**
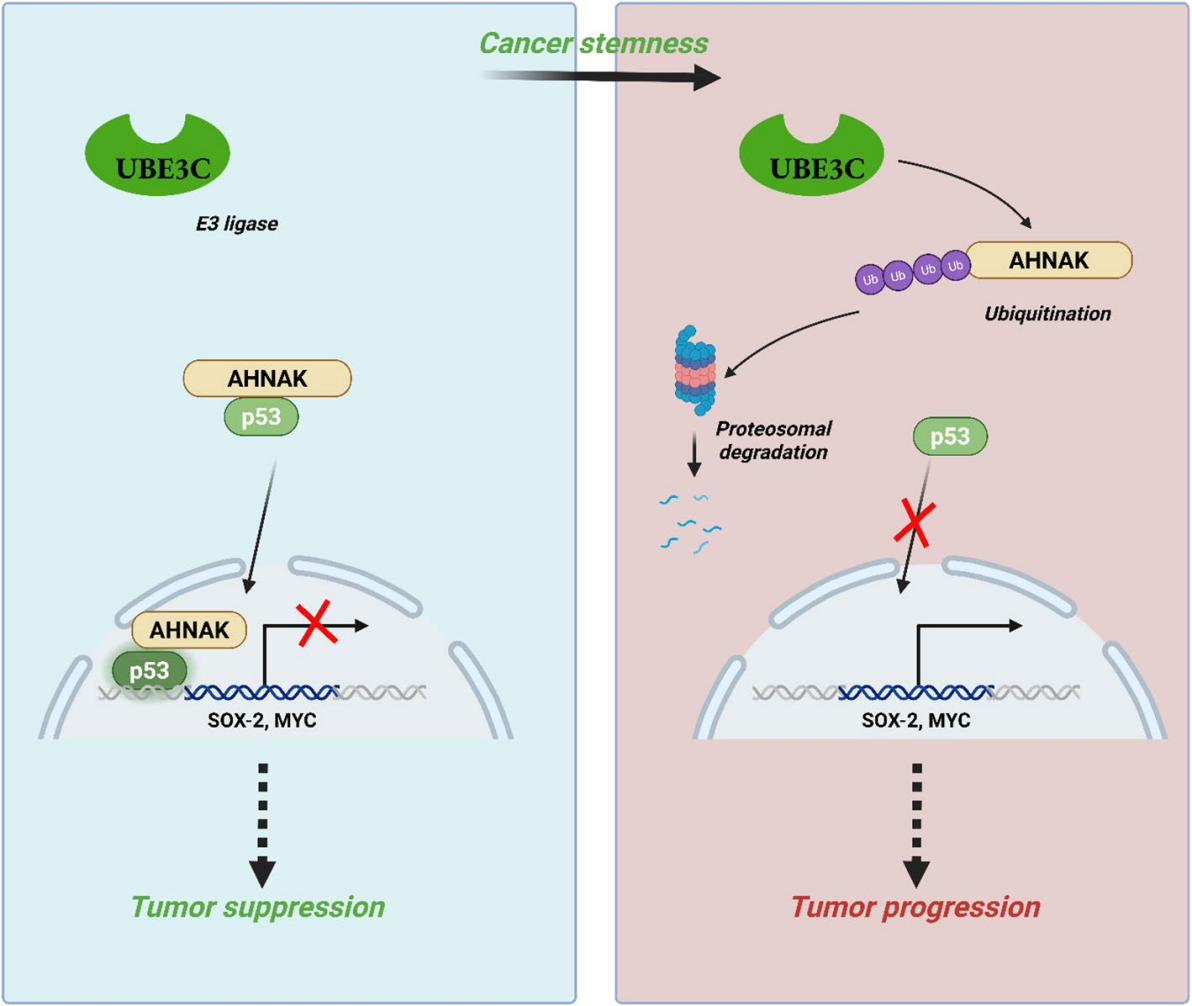
The diagram illustrates the role of UBE3C in protein regulation, showing its involvement in degrading AHNAK via ubiquitination, thereby impacting p53 activity and influencing cellular processes like stemness, shown with pathways involving SOX2 and MYC

USP44 is another notable DUB implicated in NSCLC through its regulation of the AKT signalling pathway [95]. By stabilizing PTEN, USP44 blocks the phosphorylation of AKT and subsequent targets, like mTOR and P70S6K, key drivers of cell growth and survival [96]. Zhang et al. demonstrated that a reduction in USP44 leads to decreased PTEN levels, altering AKT signalling and promoting tumor growth​ [95]. Similarly, USP49, a tumor suppressor, inhibits NSCLC growth by downregulating Cyclin D1, upregulating p53, and inducing G0/G1 cell cycle arrest, further reinforcing the importance of deubiquitination in controlling NSCLC progression​ [97]. The p53 protein, a major tumor suppressor, is also regulated by ubiquitination [98]. Gu et al. found that UBE3C, an E3 ligase, induces the breakdown of AHNAK, a key p53 regulator, facilitating tumor growth in NSCLC. Inhibiting UBE3C restores p53 activity and suppresses tumor development​ **(**Fig. 2**)** [99].

Similarly, STAT3, a key regulator of lung cancer progression, is stabilized by USP28, which enhances STAT3 signaling and promotes tumorigenesis​ [100]. Another DUB, USP41, has been identified as overexpressed in lung adenocarcinoma, where its knockdown in NSCLC cells led to decreased proliferation, enhanced apoptosis, and improved migration. This result demonstrates that USP41 could serve as a novel therapeutic target for NSCLC​ [101].

Flavoprotein monooxygenase MICAL-L2 stabilizes c-Myc and contributes in the promotion of non-small cell lung carcinoma growth as well​ [102]; MICAL-L2 facilitates NSCLC by stabilizing c-Myc. Thus, it is important to explore further how MICAL-L2 regulates cancer at the molecular level. Similarly, FBXO33 is an essential factor that regulates apoptosis and cell growth via ubiquitination. It has been demonstrated to be a lung cancer susceptibility gene, and overexpression of FBXO33 promotes protein ubiquitination-mediated apoptosis [103]. Wei et al. reported that low FBXO33 expression improves survival rates in NSCLC patients, suggesting that targeting FBXO33 could improve prognosis and treatment outcomes​ [104].

The RAF/MEK/ERK cascade is frequently activated in NSCLC due to activating mutations at different points of the cascade, such as in the RAS or BRAF gene, leading to deregulation of normal cell growth and division [105]. Similarly, DUB USP32 stabilizes the BAG3, consequently enhancing proliferation and EMT. Targeting USP32 could therapeutically benefit NSCLC therapy by targeting this pathway​ [106]. A key process for protein stability is polyubiquitination and proteasomal degradation [53]. The transcription factor TWIST1 mediates the stemness of lung cancer cells and is deubiquitinated and stabilized by USP51, which plays a stemness-maintaining role in lung carcinoma. Inhibition of USP51 could decrease the stemness of NSCLC cells and suppress their proliferation​ [107].

Another DUB, DUB3, is required for NSCLC cell-cycle progression, and it mediates cyclin A stability and changes in expression during report proliferation. Its overexpression in patients with NSCLC correlated with higher rates of recurrence and metastasis, thereby showcasing that DUB3 might be used as a potential target for antiproliferative therapy [108]. The over-expression of USP17, a DUB with a part in cell cycle control, was detected in NSCLC and correlated to poor metastasis and recurrences relapse-free survival. Targeting USP17 may function as an indicator for lung cancer metastasis​ [109]. USP14 is a special proteasome-associated DUB that trims ubiquitin chains before protein degradation by 26 S proteasome and has important roles in controlling events like cell cycle growth and apoptosis [79, 81]. In NSCLC, Yan et al. found Acf7 as a substrate of USP14, in which USP14 prevents the breakdown of the Acf7 ubiquitin pathway. They reported that silencing of USP14 inhibited cell migration.

In contrast, wild-type USP14 overexpression increased migration, suggesting a role for USP14 in NSCLC cell migration and offering new avenues for therapeutic intervention​ [110]. USP17 is another DUB involved in NSCLC [111]. USP17 is overexpressed in several cancer types, including NSCLC, and its deregulation of protein stability influences critical cellular pathways such as cell cycle progression, signalling cascades, and stress responses [108]. Zhang et al. identified that DUB USP17 is highly expressed in NSCLC and has a critical role in tumor growth. Suppression of the DUB USP17 reduced levels of cancer cell invasion and metastasis-related matrix-metalloproteases MMP3 and MMP9. Consequently, USP17 is growing as a potential target to reduce the risk of tumor development and metastasis in NSCLC​ [112]. Furthermore, Zhang et al. demonstrated that USP5 stabilized cyclin D1 in NSCLC by inhibiting its polyubiquitination, which further facilitated cell growth and colony formation. Given that USP5 inhibition induced cyclin D1 degradation and cell-cycle arrest, the targeted therapeutic intervention of the USP5-cyclin D1 axis​ target for therapy [113]. Recently more DUB inhibitors are now in preclinical or first clinical trial indicating the benefit of USP targeting in NSCLC. Proteasome-associated DUB inhibitor, VLX1570 has shown strong antiproliferative and pro-apoptotic properties in lung cancer cell models [114], with dose-limiting toxicities in a phase I trial in multiple myeloma, although unproductively [115]. In the case of USP14, genetic and pharmacological inhibition (i.e. IU1-series compounds) impedes the growth of the USP14-expressing NSCLC cells and is associated with the increase in survival in USP14-expressing tumours [116]. USP7 has also become another target of interest: selective small-molecule inhibitors, e.g. FT671 and analogs scaffolds, can inhibit USP7 activity [117], and studies of NSCLC also indicate that USP7 can maintain glycolysis through c-Abl stabilization as well as tumour growth; a definite instance of USP7 shutdown through USP7 inhibition (GNE-6776) can inhibit tumour growth and break cycles of resistance in KRAS-mutant NSCLC models [118, 119]. Collectively, these data implicate USP7 and USP14 as highly relevant clinically relevant nodes in the ubiquitin system that are undergoing drug-like molecule testing presently.

In general, USPs in NSCLC may be roughly superimposed onto individual mechanistic circuits: (i) chromatin and transcriptional regulation (USP22), (ii) checkpoint and cell-cycle regulation (USP44, USP49, USP5, DUB3), and (iii) signal-transduction and cytoskeletal remodelling (USP28, USP32, USP41, USP51, USP14, USP17). It indicates that these enzymes are not independent drivers, but a partially redundant network that hard-wires aggressive phenotypes since many of them have been found to be independently correlated with poor prognosis and metastasis. This suggests therapeutically, either strategies that aim to inhibit highly connected USPs (like USP28 or USP22) or a combination of selective USP inhibitors and direct downstream signaling node-blockers.

The E2 ubiquitin-conjugating enzyme UBE2T is also associated with NSCLC via involvement in a DNA repair pathway of FA [120]. UBE2T, another oncogenic gene with a poor prognosis in NSCLC, was identified to induce EMT and cell growth, movement, and invasion [121]. Zhang et al. observed that FANCI, a UBE2T substrate, is upregulated in NSCLC and functions as pro-tumor gene. FANCI silencing inhibited cell growth, movement, and invasion. In contrast, overexpression showed the opposite effects, indicating that targeting the UBE2T-FANCI interaction might offer a potential therapeutic option [122]. USP12 is required for the stability of proteins and to control DNA repair and transcription. USP12 dysregulation is associated with secondary cancers and represents a desirable therapeutic target in cancer [123]. In NSCLC, Chen et al. showed that USP12 stabilises RRM2, a protein required in DNA replication and tumor expansion. RRM2 was significantly upregulated in NSCLC, and its level was significantly correlated to the prognosis of NSCLC while targeting USP12-suppressed tumorigenesis in vitro. Therefore, targeting USP12 might have therapeutic benefits by interrupting many cellular pathways directly involved in cancer progression (Table 1) [124].

**Table 1:** The table summarizes key enzymes involved in NSCLC, highlighting their functions, effects on tumor progression, mechanisms, and therapeutic target potential

Enzyme	Function	Effect on NSCLC	Mechanism	Therapeutic target potential	References
USP22	Regulates H2B monoubiquitination, affects differentiation and malignancy	Linked to poor prognosis and aggressive tumors	Deubiquitination of H2Bub1 retains cancer stem-like properties	Target for inhibiting tumor proliferation, metastasis and enhancing drug sensitivity	[93]
USP22	Ubiquitin hydrolase is involved in oncogenesis and cancer progression	High levels of USP22 correlated with large tumor size and lymph node metastasis	Overexpression in NSCLC tissues, significant correlation with mRNA and protein levels, independent prognostic factor	Potential prognostic marker for early-stage NSCLC	[94]
USP44	Stabilizes PTEN, inhibits AKT phosphorylation	Inhibits tumor growth by regulating the AKT pathway	Regulates PTEN stabilization	Potential target for preventing AKT-mediated tumor progression	[95]
USP49	Tumor suppressor, regulates Cyclin D1, p53, and cell cycle arrest	Suppresses NSCLC growth and induces cell cycle arrest	Cyclin D1 and p53 regulation	A promising target for NSCLC therapy due to its role in cell cycle arrest	[97]
UBE3C	Promotes AHNAK degradation, affecting p53 regulation	Facilitates tumor growth by degrading p53 regulator AHNAK	AHNAK degradation	Target for restoring p53 activity and suppressing tumor growth	[99]
USP28	Stabilizes STAT3, promotes tumorigenesis	Enhances STAT3 signaling, promotes tumorigenesis	STAT3 stabilization	USP28 inhibition may suppress STAT3-driven tumorigenesis	[100]
USP41	Overexpressed in lung adenocarcinoma promotes proliferation	Promotes NSCLC cell proliferation and survival	Regulation of cell cycle and apoptosis	Potential target for reducing NSCLC proliferation	[101]
MICAL-L2	Stabilizes c-Myc, promotes cell proliferation	Contributes to NSCLC progression by stabilizing c-Myc	Prevents c-Myc degradation	Targeting MICAL-L2 could inhibit c-Myc-driven NSCLC growth	[102]
FBXO33	Regulates apoptosis and cell proliferation through ubiquitination	Enhances protein ubiquitination, increases lung cancer risk	Regulates apoptotic and proliferative pathways	Potential biomarker for improved prognosis and treatment outcomes	[104]
USP32	Stabilizes BAG3, promotes EMT and cell proliferation	Promotes NSCLC proliferation by enhancing RAF/MEK/ERK pathway	BAG3 stabilization	Inhibiting USP32 could disrupt EMT and proliferation pathways	[106]
USP51	Stabilizes TWIST1, maintains cancer stem-like characteristics	Maintains stem-like characteristics in NSCLC cells	TWIST1 deubiquitination	Targeting USP51 may reduce stemness and inhibit NSCLC progression	[107]
DUB3	Stabilizes Cyclin A, promotes NSCLC progression	Correlates with increased recurrence and metastasis	Cyclin A stabilization	A promising target for antiproliferative therapies	[108]
USP17	Involved in cell cycle regulation, promotes metastasis	Promotes metastasis and reduces recurrence-free survival	Inhibits cell cycle progression	Potential biomarker and target for metastatic lung cancer	[109]
USP14	Regulates protein homeostasis, promotes NSCLC cell migration	Promotes cell migration and tumor progression	Prevents degradation of substrates	USP14 inhibition could suppress cell migration and tumor spread	[110]
USP17	Regulator of cancer progression via deubiquitination	Higher USP17 expression in NSCLC cells. Knockdown of USP17 reduces cell growth, migration, invasion, and tumorigenesis.	Silencing USP17 decreases expression of matrix metalloproteases	USP17 suppression inhibits NSCLC progression	[112]
USP5	Stabilizes Cyclin D1, promotes cell proliferation	Promotes cell proliferation and colony formation	Cyclin D1 stabilization	Target for inhibiting Cyclin D1 and reducing tumor cell proliferation	[113]
UBE2T	Involved in DNA repair via FA pathway, promotes EMT and cell invasion	Stimulates proliferation and invasion through EMT	DNA repair through FANCI	Targeting UBE2T-FANCI interaction could inhibit EMT and tumor progression	[122]
USP12	Maintains protein stability, regulates DNA replication and tumor growth	Promotes tumor growth by stabilizing RRM2	RRM2 stabilization	USP12 inhibition may block tumor growth and improve prognosis	[124]

From the therapeutic perspective, opportunity and risk are visualized by the context-dependent roles of DUBs including USP9X and USP17. On the one hand, they are good targets because of their recurrent engagement in proliferation, survival, and spread. On the other, non-selective inhibition may increase genetic instability or strongly promote more aggressive clones due to the presence of tumor-suppressive or homeostatic roles in some subtypes of NSCLC. The next important step will be to delineate, on a case-by-case basis, the molecular contexts (driver mutations, co-alterations, immune microenvironment) where the net effect of each candidate DUBs can be evidently oncogenic, and to establish biomarkers by which to stratify patients prior to using DUB-directed therapies.

### Ubiquitin-regulated signaling as therapeutic nodes in NSCLC

Ubiquitin-regulated signalling pathways serve as leading players during the growth and metastasis in NSCLC, which control cell growth, survival, and movement [125]. One of the main pathways in which the Wnt/β-catenin cascade is involved in modulating cell development, differentiation, and survival [126]. The pathway serves the role of a multi-functional mechanism, and altered expression or mutations among its components have been found in various types of cancer, including NSCLC [127]. Guo et al. found that increased FGF18 and HDAC7 in NSCLC cases are linked to high tumor-grade stages and poor prognosis. HDAC7 facilitates cancer development and metastasis through the interaction with β-catenin and USP10. Reducing HDAC7’s acetylation and decreasing cancer cell activity could be an appropriate treatment for NSCLC progression, as it cannot transport bile acids to the nucleus​ [128]. One of the lessons learned during such recent work is that ubiquitination and deubiquitination is not merely downstream of these pathways but a part of the pathway as an inseparable feedback and feed-forward loop. To illustrate, the identical DUB can stabilise a receptor tyrosine kinase as well as its downstream transcription factor, thus triggering NSCLC cells into a self-reinforcing oncogenic state. It is necessary to map these loops to forecast the patients who will most probably respond to UPS-targeted interventions.

The PI3K/AKT/mTOR signaling is another central signalling cascade that controls the growth and survival of cells [129]. The tumor suppressor gene PTEN negatively modulates this pathway by dephosphorylating PIP3 to PIP2, resulting in the hindering of AKT activation [130]. PTEN is frequently lost or mutated in NSCLC, and its loss results in hyperactivation of the PI3K/AKT cascade, promoting tumorigenesis, aggressive cancer growth, poor prognosis, and tolerance to therapy [131]. He et al. showed that USP10 deubiquitinates PTEN at K63-linked polyubiquitination predominantly catalyzed by TRIM25. Knockdown of USP10 suppresses PTEN activity through the upregulation of TRIM25, which promotes tumorigenesis in NSCLC cells. The data presented indicates targeted inhibition of TRIM25 and USP10 as a novel strategy to suppress NSCLC progression [132]. The repression E3 ligase of regulation IKKα/β ubiquitination independent from AKT-p65 signalling pathway also activated NF-κB, an important transcription factor for immune responses, inflammation, and apoptosis [133]. UCHL1 caused the activation of this pathway, which resulted in the overexpression of PD-L1s, and then NSCLC cells became immune-escaped [134]. Mao et al. found that UCHL1 blocking could rescue NSCLC cell immune evasion, hence more efficacy of anti-cancer immunotherapies [135]. In addition, Zhang et al. discovered that cisplatin treatment led to upregulated USP17 and increased cisplatin resistance in NSCLC cells via PI3K/AKT signalling [136], this suggested that inhibition of USP17 may increase sensitivity to cisplatin and enhance therapeutic efficacies in NSCLC patients.

OTUD1, a DUB, is important in the stabilization of p53 and regulating proteasome pathways for protein degradation [137]. Functionally, OTUD1 has been demonstrated to act as a negative regulator of KLF4, and this has been associated with its suppression of NSCLC tumor progression [138]. Ma et al. indicated that OTUD1 was notably overexpressed in human NSCLC tissues and exerted the function of anti-tumor, but knockdown of OTUD1 may induce cancer-promoting action [139]. These results show that targeting OTUD1 could represent a novel treatment approach for NSCLC.

GIT4RA, a poorly characterized cell signaling protein, is also involved in NSCLC. Although the precise role of this intriguing molecule in vivo is still unknown, it appears to interact with G protein-coupled receptors on the cell surface. Yang et al. found that the lncRNA GIAT4RA is significantly downregulated in NSCLC and positively linked with overall survival as well as lymphatic metastasis. GIAT4RA inhibits tumorigenesis by promoting the degradation of LSH, an epigenetic guardian to preserve chromatin modification, indicating that it may be a novel tumor suppressor in NSCLC​ [140].

Another significant regulator in NSCLC is the NRF2 transcription factor that regulates explicitly cellular oxidative stress responses [141]. The preservation of mitochondrial function protects NRF2, the recycling of oxidized GSH (accumulation of reduced glutathione), and the reduction of apoptosis [142]. Although, NRF2 overexpression in NSCLC is corelated with robust tumor promotion and chemoresistance. Meng et al. found that USP11 stabilizes NRF2 levels by DUB, thereby elevating oxidative stress resistance in NSCLC cells. In addition, downregulation of USP11 induces cell ferroptosis by targeting NRF2 and provides the potential of USP11 as a treatment target to inhibit NRF2-mediated cancer growth​ [143].

Pyrroline-5-Carboxylate Reductase 3 (PYCR3) is a proline biosynthesis enzyme necessary for cellular viability and redox status [144]. Higher PYCR3 level relates to the improved viability, proliferation, and metastasis of NSCLC cells under metabolic stress. In a screen, USP9X emerged as a stabilizer of PYCR3, Becirovic et al. showed that the depletion of USP9X promoted rapid destabilization of PYCR3, which consequently blocked the proline production and restricted NSCLC development​ [145].

**Fig. 3:**
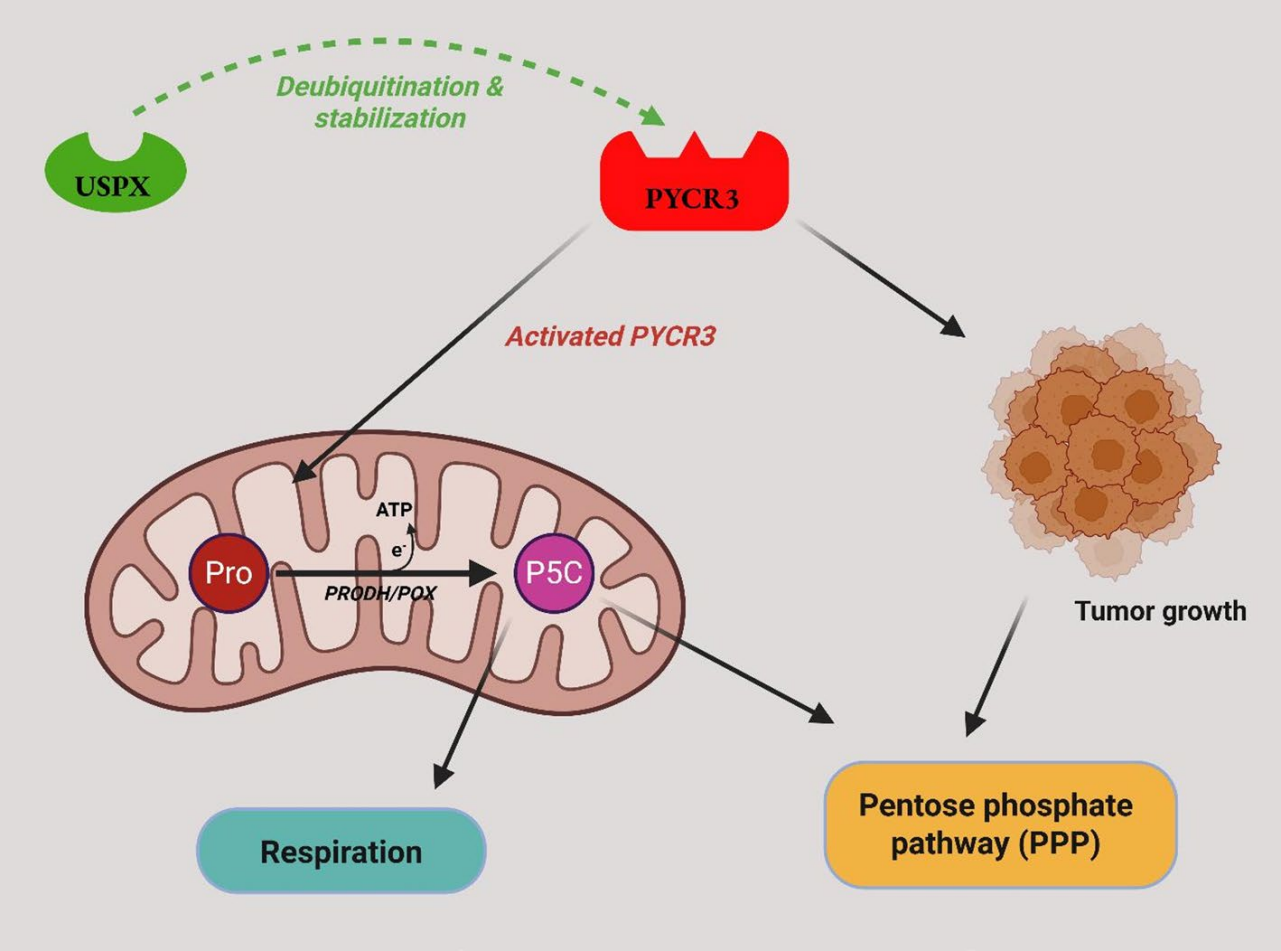
The image depicts the proline cycle’s influence on tumor growth and cellular respiration. It shows interactions between proline, PYCR3 (pyrroline-5-carboxylate reductase), USP9x, and P5C (Δ1-pyrroline-5-carboxylate), how linking the pentose phosphate pathway (PPP) to enhance tumor growth through metabolic pathways

A key cell-cycle regulator, cyclin B1, must be degraded before cells can exit from mitosis and enter into the G1 phase. This action is orchestrated through the Anaphase-Promoting Complex/Cyclosome (APC/C), a structural E3 ubiquitin ligase composed of multiple subunits [146]. This pathway is dysregulated, especially for APC11, a component of the APC/C complex that mediates the wild-type degradation of cyclin B1, in aberrant degradation leading to normal proliferation and as an oncogene-initiated event that plays essential roles in cancer development [147]. Wang et al. reported that UBA52 can interact with Cyclin B1 to control the regulatory network, and UBA52-silencing affects tumor cell proliferation in NSCLC xenografts (Fig. 3; Table 2) [148].

**Table 2:** The table summarizes key signaling pathways, enzymes, their functions, effects on NSCLC, and mechanisms involved

Pathway/protein	Enzyme	Function	Effect on NSCLC	Mechanism	References
Wnt/β-catenin pathway	USP10	Regulates cell proliferation, differentiation, and survival	Deregulation leads to excessive proliferation, abnormal tumor growth, and metastasis	Involves interaction with HDAC7, facilitated by USP10	[128]
PI3K/AKT/mTOR pathway	USP10	Regulates cell growth, proliferation, and survival	Hyperactivation promotes tumorigenesis, poor prognosis, and drug resistance	Involves PTEN dephosphorylation, reversed by USP10	[132]
AKT-p65 signalling pathway	UCHL1	Activates NF-κB, promoting immune responses and inflammation	Promotes immune escape and enhances PD-L1 levels in NSCLC cells	Stimulated by UCHL1, promoting immune escape	[135]
PI3K/AKT	USP17	Regulates tumorigenesis, invasion, and chemoresistance in NSCLC	Overexpression of USP17 increases cell proliferation and cisplatin resistance	USP17 upregulates PI3K/AKT phosphorylation, enhancing proliferation and survival in NSCLC cells	[136]
OTUD1	OTUD1	Stabilizes p53, regulates proteasome degradation pathways	Suppresses tumor progression by stabilizing tumor suppressor KLF4	Regulates protein degradation, affects KLF4 stabilization	[139]
GIT4RA	GIAT4RA	Suppresses tumorigenesis by degrading LSH, linked to chromatin modifications	Enhances degradation of proteins involved in tumorigenesis, linked to better survival	Interacts with long non-coding RNA GIAT4RA	[140]
NRF2	USP11	Manages oxidative stress and mitochondrial function	Overexpression linked to tumor growth, drug resistance	Stabilized by USP11 through deubiquitination	[143]
PYCR3	USP9X	Essential for proline biosynthesis, cell viability, and redox balance	Promotes cell survival, proliferation, and metastasis under metabolic stress	Stabilized by USP9X, it promotes proline synthesis	[145]
Cyclin B1/APC/C	APC11	Regulates cell cycle progression from mitosis to G1 phase	Dysregulation leads to unregulated cell division and cancer onset	Involves APC11, a subunit of the APC/C complex	[148]

A notable follow-up on these pathway-based approaches is that the selective degradation of NSCLC drivers can be induced with the help of proteolysis-targeting chimeras (PROTACs). The E3 ligases are then engaged by PROTACs to put oncogenic proteins on ubiquitin chains, which are degraded by proteasomes as opposed to being inhibited by enzymes [149]. Various EGFR-directed PROTACs have been created that degrade mutant EGFR (e.g. Del19) in EGFR-victimised NSCLC cell lines and xenogafts with a robust inhibition of downstream signalling and tumour growth appearing in lung cancer [150]. More recent studies are now starting to study targeted at the development of PROTACs and nano-PROTACs against additional targets related to NSCLC-signaling hubs [151–153], which postulates that inhibition of signaling hubs can provide complementary or supplementary effects in comparison with traditional kinase inhibitors.

Regardless of such therapeutic potentials, both ubiquitination and deubiquitination in NSCLC also pose serious challenges that curtail transmutation excitement. First, a number of UPS enzymes play housekeeping functions in normal tissues, and therefore, systemic consequences of inhibiting them pose dose-limiting toxicities to rapidly proliferating or heavily secretive organs. The initial observations of the efficacy of proteasome and proteasome-related DUB inhibitors in solid tumours are examples of how, despite demonstrable antitumour effects in an animal model, the therapeutic index of on-target toxicity can preclude clinical advancement. Second, ubiquitin networks entail substantial redundancy and compensatory interactions between ERP systems; therefore, inhibition against one of these E3 ligases or DUBs can be opposed by other enzymes or (by rewiring the output effectors), by other signalling nodes. Third, in some genetic or microenvironmental settings, the identical UPS element may play tumour-suppressive roles, and, therefore, non-selective inhibition may disrupt genome-maintenance programs or select more aggressive personages. These assumptions underline the fact that UPS-directed treatment in NSCLC will need a carefully chosen context, logical combinations with pathway-targeted treatments or immunotherapies, and biomarker-directed dosing schedules as opposed to a one-size-fits-all model.

### Role of enzymes in resistance mechanisms of NSCLC

Multi-drug resistance emergence is one of the most clinically significant implications of UPS dysregulation of NSCLC. In this case, ubiquitin-related enzymes play dynamic buffering functions enabling tumour cells to withstand genotoxic, metabolic, and immunological insults posed by therapy. In lieu of defining radioresistance, chemoresistance, and targeted-therapy resistance as distinct phenomena, it is useful to note that a rather large list of enzymes commonly occurs in recent studies (UCHL1, USP14, USP35, OTUD5) across these conditions.

Radiotherapy resistance continues to pose a major obstacle in the therapeutic management of NSCLC, necessitating new strategies to improve cancer cell responsiveness to radiation [154, 155]. The UPS is crucial in controlling cancer progression and treatment response, though its exact role in NSCLC resistance was initially unclear [156]. Tang et al. found that targeting UBB and UBC increased radiosensitivity, facilitated apoptosis, and suppressed NF-κB nuclear translocation, thereby repressing both cancer progression and radioresistance in NSCLC xenografts. This underscores the potential of targeting ubiquitin as an innovative treatment approach for NSCLC​ [157].

Chemoresistance is another major challenge in treating NSCLC [158, 159]. Ding et al. identified UCHL1 as overexpressed in pemetrexed (PEM)-resistant NSCLC cells, contributing to poor patient outcomes. UCHL1 promotes resistance by upregulating thymidylate synthase, a key enzyme that reduces DNA damage caused by PEM and prevents arrest of the cell cycle. Silencing or inhibiting UCHL1 overcomes PEM resistance, suggesting that UCHL1 emerges as a valuable focus for overcoming drug resistance in NSCLC​ [160].

Doxorubicin and cisplatin resistance further complicate NSCLC treatment. These drugs work by inhibiting topoisomerase II and inducing DNA cross-links, respectively. Still, cancer cells often develop mechanisms to evade their effects, including the upregulation of ATP-binding cassette transporters and more efficient DNA repair systems [161]. Kang et al. discovered that the DUB OTUD5 is highly downregulated in NSCLC tissues, leading to poor prognosis. Knockdown of OTUD5 decreases the stability of tumor suppressors such as p53 and PDCD5, increasing cancer cell proliferation, migration, and resistance to doxorubicin and cisplatin. These findings suggest that restoring OTUD5 function could reduce chemoresistance by reactivating apoptosis-related pathways​ [162]. USP35, which is part of the USP family, is involved in regulating mitophagy, cell division, and cancer cell resistance [163]. Liu et al. revealed that USP35 stabilizes BIRC3 through deubiquitination in NSCLC cells, contributing to cisplatin resistance. Downregulation of USP35 enhances the apoptotic response to cisplatin, while overexpression decreases apoptosis, implying that targeting USP35 may offer a viable approach for addressing cisplatin resistance​ [164].

MicroRNA is a critical molecule that regulates drug response and cell survival mechanisms that are involved in cancer resistance [165]. Yu et al. reported that hsa-miR-124a was one of the downregulated miRNAs in NSCLC and was targeting USP14. Consequently, NSCLC patients who express lower levels of hsa-miR-124a and have USP14 inwards tend to have a worse prognosis. Restoration of hsa-miR-124a expression inhibits tumorigenesis and stemness and sensitizes cells to gefitinib. The results thus implied that hsa-miR-124a might be employed as a potential biomarker for NSCLC diagnosis and as an effective target of USP14 inhibition​ [166].

The enzyme Fruitage Binding Protein 1 (FBP1) plays a tumor-suppressive role in cancer by regulating glucose metabolism and the switch between glycolysis and gluconeogenesis. FBP1 interacts with cellular stress responses and apoptosis pathways, which helps tumor cells adapt to adverse conditions [167]. FBP1 suppresses NSCLC stemness and tumorigenicity by inducing ubiquitin-proteasomal degradation of NICD1. This metabolic shift disrupts glucose homeostasis, affecting cancer cell survival and metastatic potential. The study suggests breaking the FBP1-FBXW7-NICD1 axis could contribute to NSCLC pathogenesis and offer potential therapeutic options (Table 3) [168]. Collectively, the resistance-linked UPS alterations in NSCLC characterize a contributing philosophy: the enzymes that buffer the acute stress during treatment are harness into assisting the persistent contentment of tumor growth in the long run. According to this view, it is plausible that effective resensitization concepts will entail temporally synchronized interventions, such as transient inhibition of particular DUBs or E3 ligases throughout chemotherapy or radiotherapy, or rationally combinable ones with immune checkpoint blockades to ensure that there is no emergence of highly stress-adapted, immune-evasive clones.

**Table 3:** This table highlights enzymes/proteins involved in NSCLC resistance, showing their effects on radiosensitivity, chemoresistance, and tumor progression mechanisms

Enzyme/protein	Function	Effect on NSCLC	Mechanism	References
UBB/UBC	Regulates radiosensitivity and cancer progression	↑ Radiosensitivity, ↑ Apoptosis, ↓ NF-κB nuclear translocation	Increases radiosensitivity by facilitating apoptosis and suppressing NF-κB	[157]
UCHL1	Upregulates thymidylate synthase, reducing DNA damage	↑ Resistance to pemetrexed and other chemotherapeutic agents	Reduces PEM-induced DNA damage, prevents cell cycle arrest	[160]
OTUD5	Regulates tumor suppressors such as p53, promotes apoptosis	↓ OTUD5 expression linked to ↓ Prognosis and ↑ Resistance to doxorubicin and cisplatin	Regulates tumor suppressors like p53 and PDCD5	[162]
USP35	Stabilizes BIRC3, contributes to cisplatin resistance	↓ USP35 enhances apoptotic response, ↑ Cisplatin resistance with overexpression	Stabilizes BIRC3 through deubiquitination	[164]
USP14	Targets hsa-miR-124a, regulates drug response and survival	↓ hsa-miR-124a linked to ↓ Survival, ↑ Gefitinib sensitivity when restored	Regulates drug response and survival via miRNA downregulation	[166]
FBP1	Regulates glucose metabolism and promotes NICD1 degradation	↓ Stemness and tumorigenicity by disrupting glucose homeostasis	Promotes NICD1 degradation, disrupts glucose homeostasis	[168]

**Fig. 4:**
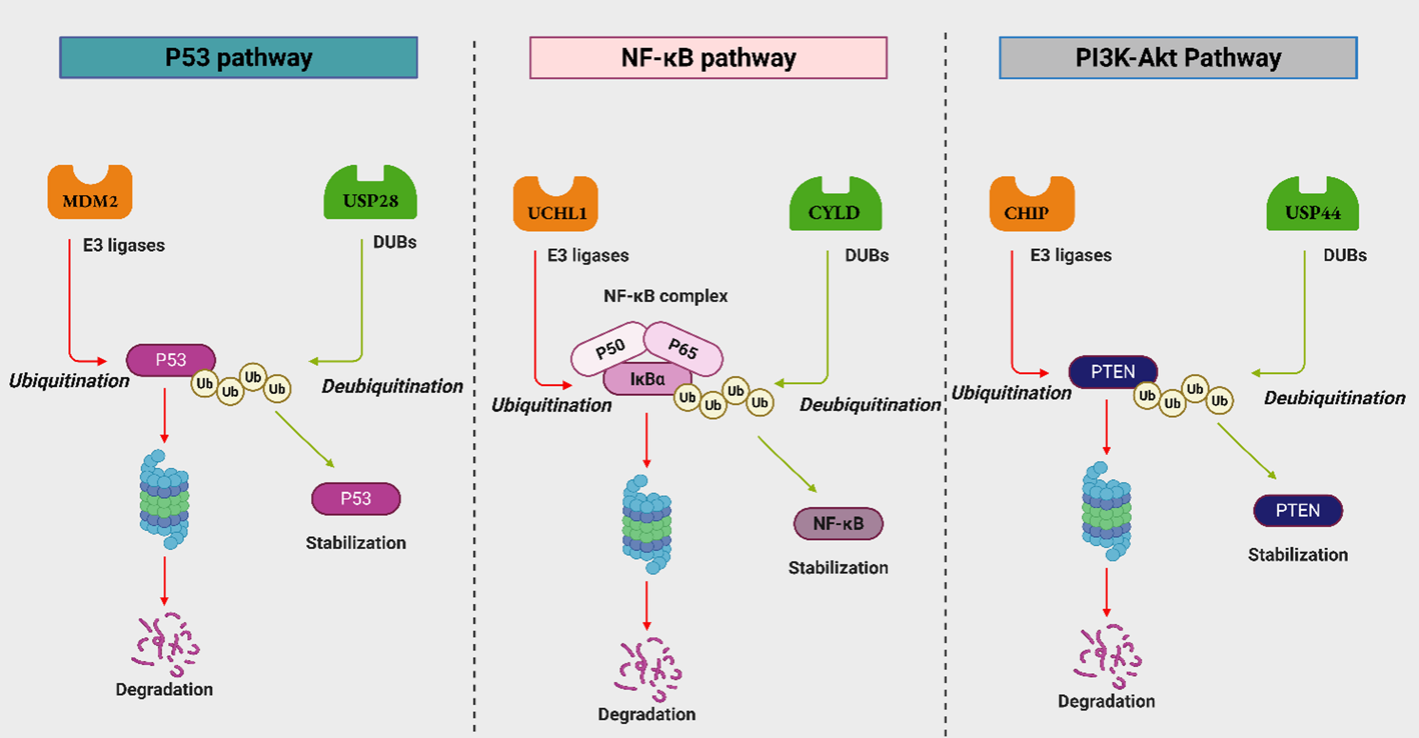
Mechanistic convergence of E3 ligases and DUBs on oncogenic and tumor suppressor pathways in NSCLC. This model illustrates how dysregulation of the Ubiquitin-Proteasome System (UPS) drives NSCLC by converging on three major signaling axes (p53, NF-κB, PI3K/AKT/mTOR)

In Sect. 5.1–5.3, a number of ubiquitin-modifying enzymes arise as largely oncogenic drivers in NSCLC. They are classified as a variety of USPs (USP22, USP28, USP32, USP41, USP51, DUB3, USP17 and USP14), E2 and E3 enzymes (UBE2T, MICAL-L2, Skp2) and metabolic or signalling regulators that are regulated by ubiquitination (NRF2, PYCR3 and cyclin B1). All these combines to drive crucial malignant phenotypes, such as sustained proliferation, upgraded survival signalling, epithelial mesenchymal transition, stemness, metastatic spread and radiotherapy resistance and chemotherapy resistance. Their frequent overexpression or hyperactivation of NSCLC combined with their high negative correlations with good prognosis justifies them as oncogenic UPS components and provides the basis on which they should be targeted with inhibitors, PROTAC-mediated degradation, and as part of combination regimens. Additionally, the mechanistic convergence of E3 ligases and DUBs on oncogenic regulation of NSCLC by converging on three major signaling axes (p53, NF-κB, PI3K/AKT/mTOR) was depicted in Fig. 4.

## Challenges and future directions

The mechanistic evidence overviewed in the sections above all lead to our core hypothesis that dysregulated ubiquitination and deubiquitination represent a systems-level centre in NSCLC. Numerous E3 ligases and DUBs interact with shared substrates and pathways and organise proliferation adoption and therapy resistance in a single regulatory layer.

Ubiquitination and deubiquitination are general regulators in receptor signalling, cell-cycle regulation, DNA-damage response, and metabolism with various E3 ligases and DUBs interacting with multiple signaling substrates and alternate choice of ubiquitin chain type subsequently determining degradation, recycling, or sorting into signaling cascades that determine the NSCLC growth and therapeutic response. However, there are still significant gaps, because DUBs (USP9X and USP17) have context-dependent, even opposite roles, and the role played by ubiquitin-dependent versus ubiquitin-independent resistance mechanisms is still not fully understood, especially in immunotherapy and future-generation targeted therapies. To resolve these gaps, there should be systematic mapping of E3/DUB-substrate interaction that are intertwined with stress-response pathway and validated in model systems that recapitulate tumour heterogeneity to make mechanistically informed, biomarker-directed treatment approaches possible.

**Fig. 5:**
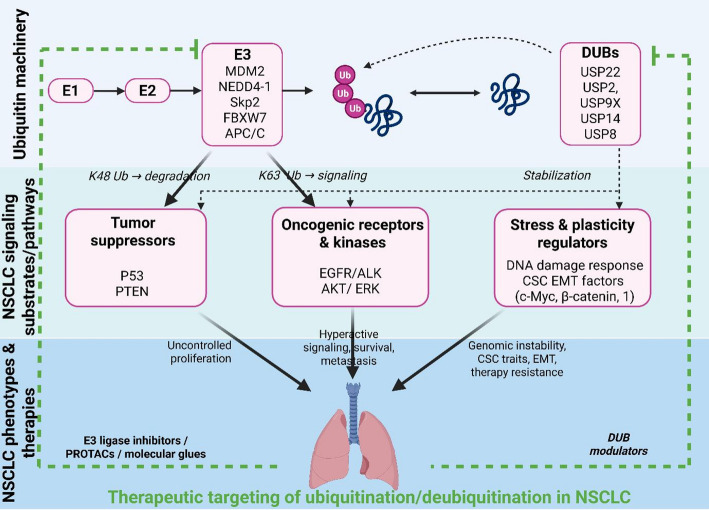
Overview of ubiquitination and deubiquitination modulator mechanisms in NSCLC progression and therapeutic strategies

Even with these significant advancements, the translation of ubiquitination biology into NSCLC therapeutics is limited by several conceptual and practical gaps. Research on the functions of ubiquitination and deubiquitination in NSCLC is limited, particularly in the crosstalk of ubiquitination pathways with other cellular signalling mechanisms like autophagy, apoptosis, and DNA damage response [49]. There is also limited data on the involvement of many DUBs in the development of NSCLC, including USP28 and USP17 [67, 109]. Other enzymes, including less-studied DUBs, have not been thoroughly investigated for their specific roles in different NSCLC subtypes and disease stages [18]. It is unclear what their compensatory potential and crosstalk might be and how they can affect resistance to existing therapies. This predicts that the response to many available therapies will be imprecise and will complicate the development of new ones. Overview of ubiquitination and deubiquitination modulator mechanisms in NSCLC progression and therapeutic strategies were depicted in Fig. 5.

Another complication is level of complexity which exists as many UPS components show subtype- and driver-context specificity in NSCLC. As an illustration, enzymes that regulate receptor tyrosine kinase signalling the most could be critical in EGFR- or ALK-driven adenocarcinomas, but ligases and DUBs which incorporate inflammatory or metabolic pressure could be preferentially used in smoking-associated or squamous tumours. Concurrently, network-level rewiring is often part of adaptive resistance to specific agents or immunotherapy due to the replacement of the repressed node by different circuits of E3/DUB interactions or coplanar pathways of stress response. The following insights suggest that future UPS-targeted strategies must not merely focus on the presence or absence of a particular enzyme, but instead the overall mutational state, histological subtype, and treatment history and hopefully by use of systems-biology and multi-omics-based methods, genuinely subtype-specific vulnerabilities.

The alteration of ubiquitination or deubiquitination pathways presents potential for innovative treatment approaches in NSCLC [156]. Small chemical activators or inhibitors targeting ubiquitin ligases or DUBs implicated in NSCLC progression and resistance can be developed. USP-targeting inhibitors can improve oncoprotein degradation and impede tumor development [169]. Ubiquitination-modifying medications can be combined with other therapies to increase effectiveness and overcome resistance. CRISPR-Cas9 gene-editing technology can disrupt aberrant ubiquitination mechanisms, specifically knocking out or changing genes encoding for specific ubiquitin ligases or DUBs [170]. The use of proteolysis-targeting chimeras (PROTACs) and the UPS to target undruggable proteins in NSCLC presents a promising strategy [171]. Combining systems biology and computer modeling could help understand the intricate networks controlling ubiquitination and deubiquitination [172], leading to new therapeutic targets and pharmacological reaction forecasting.

## Conclusion and future perspective

Ubiquitination and deubiquitination pathways are essential regulators of protein stability and function and play a central role in NSCLC pathophysiology and treatment resistance. These modifications, which are regulated by a balance of DUBs and ubiquitin ligases, directly impact cell cycle regulation, apoptosis, and DNA repair. The most common property within the context of NSCLC is that cancer cells grow uncontrollably and are capable of developing resistance to the therapeutic agents due to the deregulation in these avenues. USP28, USP17, and USP14 enzymes are key players in these activities as they stabilize oncoproteins and modulate signalling pathways that drive the development of tumors and resistance. The enzymes could be a target for the development of improved NSCLC therapy. However, more research is required to understand how ubiquitination pathways interact with other biological functions, potentially leading to novel therapeutic targets and inventive remedies. Techniques to target dysregulated ubiquitination processes include PROTACs, gene-editing technologies, and small chemical inhibitors.

## Data Availability

No datasets were generated or analysed during the current study.
